# Development and validation of multiplex one-step qPCR/RT-qPCR assays for simultaneous detection of SARS-CoV-2 and pathogens associated with feline respiratory disease complex

**DOI:** 10.1371/journal.pone.0297796

**Published:** 2024-03-22

**Authors:** Côme J. Thieulent, Mariano Carossino, Laura Peak, Wendy Wolfson, Udeni B. R. Balasuriya

**Affiliations:** 1 Louisiana Animal Disease Diagnostic Laboratory, School of Veterinary Medicine, Louisiana State University, Baton Rouge, Louisiana, United States of America; 2 Department of Pathobiological Sciences, School of Veterinary Medicine, Louisiana State University, Baton Rouge, Louisiana, United States of America; 3 Department of Veterinary Clinical Sciences, School of Veterinary Medicine, Louisiana State University, Baton Rouge, Louisiana, United States of America; Universidad Cooperativa de Colombia, COLOMBIA

## Abstract

Feline respiratory disease complex (FRDC) is caused by a wide range of viral and bacterial pathogens. Both Influenza A virus (IAV) and Severe Acute Respiratory Syndrome Coronavirus-2 (SARS-CoV-2) also induce respiratory diseases in cats. Two one-step multiplex qPCR/RT-qPCR assays were developed and validated: FRA_1 (Feline respiratory assay 1) for the detection of four viral targets and FRA_2 for the detection of three bacteria associated with FRDC. Both multiplex assays demonstrated high specificity, efficiency (93.51%–107.8%), linearity (> 0.998), analytical sensitivity (≤ 15 genome copies/μl), repeatability (coefficient of variation [CV] < 5%), and reproducibility (CV < 6%). Among the 63 clinical specimens collected from FRDC-suspected cats, 92.1% were positive for at least one pathogen and co-infection was detected in 57.1% of samples. *Mycoplasma felis* (61.9%) was the most found pathogen, followed by feline herpesvirus-1 (30.2%), *Chlamydia felis* (28.7%) and feline calicivirus (27.0%). SARS-CoV-2 was detected in two specimens. In summary, this new panel of qPCR/RT-qPCR assays constitutes a useful and reliable tool for the rapid detection of SARS-CoV-2 and viral and bacterial pathogens associated with FRDC in cats.

## Introduction

Feline respiratory disease complex (FRDC) is a contagious respiratory or ocular disease caused by one or multiple viral and bacterial pathogens. FRDC is a major cause of morbidity and mortality in cats, particularly in high density facilities, such as shelters [1]. While FRDC may occur in adult cats, kittens are prone to develop more severe clinical signs [2]. Common clinical manifestations include mucopurulent nasal discharge, sneezing, conjunctivitis and ocular discharge, coughing, fever, lethargy and inappetence of varying severity [3]. FRDC is the result of a complex multifactorial interaction between respiratory pathogens, stress, and individual animal susceptibility [2–4]. A wide array of viruses and bacteria are reported to induce FRDC in cats. The two most prevalent viruses responsible for FRDC are feline herpesvirus-1 (FHV-1; *Varicellovirus felidalpha1*, the causative agent of feline viral rhinotracheitis [FVR]) and *feline calicivirus* (FCV). The bacteria *Bordetella bronchiseptica*, *Chlamydia felis* and *Mycoplasma felis* are also frequently detected in cats with FRDC [3–11]. Co-infections are also common [3, 12, 13].

In the last two decades, Influenza A viruses (IAV: *Alphainfluenzavirus influenzae*) and *Severe acute respiratory syndrome-related coronavirus 2* (SARS-CoV-2) have been identified as emerging infections in cats [14]. Recent outbreaks of IAV infection have been reported [15, 16], and five subtypes of IAV were identified as the cause of acute respiratory illness including H5N1 [17–22], H1N1 [23–27], H5N6 [28–30], H7N2 [31, 32] and H3N2 [33, 34]. Depending on the IAV subtype, clinical signs in cats may be subclinical, or develop mild to severe and occasionally lethal respiratory disease [16, 20]. IAV seems to be transmitted to cats by birds, humans, and dogs [16, 33–36]. Furthermore, during COVID-19 pandemic dogs, cats and many other animal species were infected with SARS-CoV-2 [37–39]. Cats are susceptible to SARS-CoV-2 following both experimental and natural infection, although natural cases have been only sporadically reported [39–49]. Large wild felids including lions, tigers and pumas are also susceptible to natural infection [50–53]. SARS-CoV-2-infected cats may remain asymptomatic or display signs of respiratory disease clinically indistinguishable from other respiratory pathogens. [14, 40, 43, 44, 54, 55]. Based on the public health importance of SARS-CoV-2 and IAV (i.e., zoonotic potential), rapid laboratory diagnosis to differentiate these two infections from other common viral and bacterial agents is critical in controlling outbreaks and implementing appropriate public health measures.

Multiplex qPCR and RT-qPCR assays are commonly used in veterinary diagnostic settings [56–62]. Multiplexing has several advantages including reduction of cost, reduction in the amount of clinical sample needed, reduction of set-up and analysis time, and finally improved precision by minimizing pipetting errors. In this study, we developed and evaluated the analytical performance of a panel of two multiplex one-step qPCR/RT-qPCR assays for simultaneous detection and differentiation of viruses (Feline Respiratory Assay_1: FRA_1) and bacteria (FRA_2) associated with FRDC in cats. This new panel was then used to test clinical specimens collected from FRDC-suspected felines in Louisiana, USA, between 2020 to 2022. Overall, this newly developed highly sensitive panel of multiplex qPCR/RT-qPCR assays can simultaneously detect all FRDC associated pathogens, as well as IAV and SARS-CoV-2 in feline clinical specimens with high analytical sensitivity and specificity.

## Materials and methods

### Viruses and bacteria

The panel of reference pathogens (viruses and bacteria) used for evaluating specificity (inclusivity/exclusivity) of each qPCR and RT-qPCR assay in singleplex and in multiplex format is presented in Table 1. RNA derived from Canine Influenza A (CIV) H3N2 VSL-1355 and CIV H3N8 A/Ca/FL/15592/04 were kindly provided by Dr. Diego Diel and Dr. Edward Dubovi (Department of Population Medicine and Diagnostic Sciences, Cornell University College of Veterinary Medicine, Ithaca, NY), respectively. All other prototype strains were obtained from the American Type Culture Collection (ATCC^®^; Manassas, VA), or BEI Resources (Manassas, VA).

**Table 1:** Panel of viruses and bacteria associated with feline respiratory disorders, related pathogens and SARS-CoV-2 variants used to assess the specificity of each qPCR/RT-qPCR assay.

Pathogens	Reference strain	Source
Feline Calicivirus (FCV)	VR-782^™^	ATCC^®^
SARS-CoV-2 USA-WA1/2020	NR-52281	BEI Resources
SARS-CoV-2 Alpha (B.1.1.7)	NR-54020	BEI Resources
SARS-CoV-2 Beta (B.1.351)	NR-55282	BEI Resources
SARS-CoV-2 Delta (B.1.617.2)	NR-55671	BEI Resources
SARS-CoV-2 Omicron (B.1.1.529)	NR-56461	BEI Resources
Feline Herpesvirus 1 (FHV-1)	VR-814^™^	ATCC^®^
Canine Influenza A (CIV) H3N2	VLS-1355	Cornell University^a^
CIV H3N8	A/Ca/FL/15592/04	Cornell University^b^
*Bordetella bronchiseptica* E014	NR-44164	BEI Resources
*Chlamydia felis*Everett et al.	VR-120^™^	ATCC^®^
*Mycoplasma felis*Cole et al.	23391^™^	ATCC^®^
*Mycoplasma cynos* Rosendal	27544^™^	ATCC^®^
*Mycoplasma canis*	NR-3865	BEI Resources
Feline Coronavirus (FCoV)	L1911562	LADDL^c^
Feline Infectious Peritonitis Virus (FIPV)	NR-43287	BEI Resources
Feline Panleukopenia Virus (FPlV)	VR-648^™^	ATCC^®^

^a^Kindly provided by Dr. Diego Diel

^b^Kindly provided by Dr. Edward Dubovi

^c^LADDL: Louisiana Animal Disease Diagnostic Laboratory, School of Veterinary Medicine, Louisiana State University, Louisiana, Inited States of America

### Clinical specimens

A total of 63 clinical specimens from 39 FRDC-suspected felines that were submitted for routine diagnostic testing to the Louisiana Animal Disease Diagnostic Laboratory (LADDL) between 2020 and 2022 were included in this study (S1 Table). The specimens were submitted by practicing veterinarians or the attending veterinarian from the LSU School of Veterinary Medicine Shelter Medicine Program. The cats submitted through the Shelter Medicine Program came from four shelters (Shelter 1 to 4) located in and around Baton Rouge, LA, in 2022. Nasal and pharyngeal specimens were collected using sterile oropharyngeal/nasal swabs (VMRD, Pullman, WA) and resuspended in either 2 ml of BHI Broth (Hardy Diagnostics, Santa Maria, CA) or 2 ml of PrimeStore^®^ molecular transport medium (VMRD) and stored at 4°C until used.

### Nucleic acid extraction

Nasal swab samples were vortexed, spun down and total nucleic acid were extracted using the taco^™^ mini nucleic acid automatic extraction system (GeneReach, Taichung, Taiwan) following manufacturer’s recommendations. One-hundred microliters of nasal swab suspensions were extracted and eluted in the same volume of elution buffer. The extracted nucleic acid samples were stored at -80°C until used.

### Primers probes design

Specific forward and reverse primers and probes targeting the glycoprotein B (gB) of FHV-1 and open reading frame (ORF) 1 of FCV were designed using IDT’s PrimerQuest tool (https://www.idtdna.com/Primerquest/home/Index) from sequences available on the GenBank nucleotide database (https://www.ncbi.nlm.nih.gov/nuccore/) (Table 2). The primers and probe sequence specificity were further validated *in silico* using the NCBI Basic Local Aligment Search Tool (BLAST; https://blast.ncbi.nlm.nih.gov/Blast.cgi?PROGRAM=blastn&PAGE_TYPE=BlastSearch&LINK_LOC=blasthome). Self-annealing sites, hairpin loop formation and 3’ complementarity were verified using IDT’s OligoAnalyzer tool (https://www.idtdna.com/calc/analyzer). Influenza A virus [63] primers and probe targeting the matrix (M) gene were used as previously published [63, 64] with addition of degeneracies in the primers sequences in order to match that of IAV sequences from cats available on the Influenza Research Database (https://www.fludb.org) (Table 2). Sequences of primers and probes for SARS-CoV-2 (US CDC SARS-CoV-2 N1 assay; Lu et al., 2020), *Bordetella bronchiseptica* [66], *Mycoplasma felis* [67] and *Chlamydia felis* [68] were used as previously published (Table 2).

**Table 2:** Primers and probe sequences used for the detection of FRDC-associated pathogens and SARS-CoV-2.

Target (gene)	Oligonucleotide ID	Primers and probe sequences (5’-3’)	Nucleotide position	Product size (bp)	GenBank accession #	Reference
SARS-CoV-2 (Nucleocapsid [ORF9a])	SARS-CoV-2_N1-F	GACCCCAAAATCAGCGAAAT	28,287–28,306	72	MN985325.1	[63]
	SARS-CoV-2_N1-R	TCTGGTTACTGCCAGTTGAATCTG	28,358–28,335			
	SARS-CoV-2_N1-P	FAM-ACCCCGCATTACGTTTGGTGGACC-QSY	28,309–28,332			
Influenza A virus (Matrix protein)	IAV_M-F	AGATGAG**Y**CTTCTAACCGAGGTCG	24–47	101	MF978391.1	[61, 62] with modifications
	IAV_M-R1^a^	TGCAAAGACATCTTCAAGT**Y**TCTG	124–101			
	IAV_M-R2^a^	TGCAAAGACACTTTCCAGTCTCTG	124–101			
	IAV_M-P	VIC-TCAGGCCCCCTCAAAGCCGA-QSY	74–93			
Feline calicivirus (ORF-1)	FCV_ORF1-F	CCGCCAATCAACATGTGGTA	2,427–2,446	114	L40021.1	This article
	FCV_ORF1-R	GCACATCATATGCGGCTCTG	2,540–2,521			
	FCV_ORF1-P	ABY-TGATTTGGCCTGGGCTCTTCG-QSY	2,464–2,484			
Feline herpesvirus 1 (Glycoprotein B [UL27])	FHV-1_gB-F	GTTAATCCCGACGATCCGTTAC	77,404–77,425	101	MH070348.1	This article
	FHV-1_gB-R	CAGGGACACAGTGGCTATTT	77,504–77,485			
	FHV-1_gB-P	Cy5-CTACTCGGT/TAO/ATTGCAGCGACTGGC-3IAbRQSp	77,459–44,436			
*Bordetella bronchiseptica* (Intergenomic region between flaA and fliA B)	Fla2-F	AGGCTCCCAAGAGAGAAAGGCTT	1,140,858–1,140,880	118	CP019934.1	[64]
	Fla12-R	AAACCTGCCGTAATCCAGGC	1,140,975–1,140,956			
	Fla-P	FAM-ACCGGGCAGCTAGGCCGC-QSY	1,140,887–1,140,904			
*Mycoplasma felis* (tuf)	Mfelis_tuf-F	TAAATTAGCTCTTGATGGTGTTCCT	469–493	100	FJ896389.1	[65]
	Mfelis_tuf-R	TTCAAAGTCTTTTTCTGGAGTTTCA	568–544			
	Mfelis_tuf-P	VIC-TGAGAAGAAAAAGTTATGGAATTAATGGATGCA-QSY	497–529			
*Chamydia felis* (ompA)	Cfelis_ompA-F	TCGGATTGATTGGTCTTGCA	449–468	78	AY184290.1	[66]
	Cfelis_ompA-R	GCTCTACAATGCCTTGAGAAATTTC	526–502			
	Cfelis_ompA-P	ABY-ACTGATTTCGCCAATCAGCGTCCAA-QSY	472–496			

^a^IAV_M-R1 and IAV_M-R2 were used at equimolar amount (200 nM).

3IAbRQSp: 3’ Iowa Black^®^ RQ; ABY, ABY^™^ dye; Cy5: Cyanine-5 dye; F: forward primer; FAM, 6-carboxyfluorescein dye; P: probe; QSY, QSY^™^ quencher; R: reverse primer; TAO: TAO^™^ quencher; VIC, VIC^™^ dye.

### Multiplex TaqMan^®^ quantitative PCR (qPCR) and reverse transcription PCR (RT-qPCR) assays for feline respiratory pathogens

A 4-plex RT-qPCR assay and a 3-plex qPCR assay were developed and designated as FRA_1 (targeting SARS-CoV-2, IAV, FCV and FHV-1) and FRA_2 (targeting *B*. *bronchiseptica*, *M*. *felis* and *C*. *felis*). Both assays were performed in a total volume of 25 μl containing 12.5 μl of 2X QuantiTect^™^ Multiplex RT-PCR Master Mix, 0.25 μl of QuantiTect^™^ RT Mix (QIAGEN, Hilden, Germany), 1.25 μl of primers (200 nM) and fluorogenic probes (200 nM) mix, 6 μl of RNase free water and 5 μl of template DNA/RNA. All reactions were run on a 7500 Fast Real-Time PCR System (Applied Biosystems, Waltham, MA) with the following thermal profile: a reverse transcription step (20 min at 50°C) following by an initial activation step (15 min at 95°C) and 40 cycles of denaturation & annealing/extension (45 sec at 94°C & 75 sec at 60°C). The complete step-by-step protocol has been deposited on the protocols.io platform (https://dx.doi.org/10.17504/protocols.io.ewov1q4z2gr2/v1).

### Synthesis of *in vitro* transcribed RNA and DNA

Specific plasmid DNA and *in vitro* transcribed (IVT) RNA were synthesized in order to determine the analytical sensitivity of each multiplex qPCR/RT-qPCR assay as previously described [56], with minor modifications. Two inserts, containing the target regions of each assay flanked by *PstI* and *HindIII* restriction sites, were chemically synthesized and cloned into the pGEM^®^-3Z vector (Promega, Madison, WI) downstream the T7 promoter (pGEM−3Z_FCV_FHV1_IAV_SCoV2 and pGEM−3Z_Bbron_ Mcynos_Mfelis_Cfelis) by GeneArt Gene Synthesis (Thermo Fisher Scientific, Waltham, MA). Transformed *Escherichia coli* DH10β cells were incubated overnight at 37°C with agitation (270 rpm). Plasmid DNA was extracted using the QIAprep Spin Miniprep kit (QIAGEN). Both plasmids were linearized using *HindIII* restriction enzyme and plasmid DNA concentration was measured using Qubit dsDNA BR Assay Kit (Thermo Fisher Scientific). pGEM−3Z_FCV_FHV1_IAV_SCoV2 plasmid was subjected to *in vitro* transcription using the Megascript^®^ T7 Transcription Kit (Thermo Fisher Scientific) following manufacturer’s recommendations. Subsequently, DNase treatment was performed with TURBO^™^ DNase (Thermo Fisher Scientific) for 15 min at 37°C. The IVT RNA products were purified using MEGAclear^™^ Transcription Clean-Up Kit (Thermo Fisher Scientific) and quantified using Qubit RNA BR Assay Kit (Thermo Fisher Scientific). The number of plasmid DNA and IVT RNA copies/μl were calculated according to the following formula [56, 60–62, 69–72]:

$$\begin{array}{c} \text{Number}\;\text{of}\;\text{plasmid}\;\text{DNA}\;\text{and}\;\text{IVT}\;\text{RNA}\;\text{molecules}/\mu l=\\ \frac{Avogadro^{'}snumber\;\left(6.022\;\times \;10^{23}\right)\;\times \;Plasmid\;DNA/IVTRNA\;concentration\;(\frac{g}{\mu l})}{Plasmid\;DNA/IVT\;RNA\;molecular\;weight\;(\frac{g}{mol})} \end{array}$$

Plasmid DNA and IVT RNA molecular weight were calculated using Molbiotools website (https://molbiotools.com/dnacalculator.php) and concentrations were adjusted to 10^7^ copies/μl in nuclease-free water containing 40 ng/μl of yeast tRNA (Thermo Fisher Scientific) and stored at -80°C until use.. Ten-fold serial dilutions of plasmid DNA/IVT RNA was directly used for determining the analytical sensitivity of the qPCR assay targeting the DNA viruses and bacteria.

### Analytical parameter determination and statistical analysis

Analytical parameters were determined as previously described [56] with minor modifications. Standard curves were generated using a ten-fold dilution series of plasmid DNA or IVT RNA (10^7^ to 10^2^ copies/μl) in triplicate. Coefficients of determination (R^2^) were used to assess curve fitness. Amplification efficiency [E (%)] was calculated after regression analysis using the following formula: E = [10^−1/slope^ -1] × 100. Limit of detection with 95% confidence (LOD_95%_) of each assay was determined by statistical probit analysis (non-linear regression model) using SPSS 14.0 software (SPSS Inc., Chicago, IL) from twelve replicates per dilution ranging from 10^3^ to 10^0^ copies/μl. Cycle threshold (Ct) cut-off values were determined using the following formula: Ct cut-off = Average replicate values of the endpoint dilution + (3 × standard deviation (SD) [73]. Intra-run and inter-run imprecision were determined by performing 12 replicates on the same run or three replicates on two independent runs of plasmid DNA/IVT RNA containing 10^5^ to and 10^3^ copies/μl, respectively. The coefficient of variation (%CV) was calculated using the following formula: %CV = 100 × (standard deviation of replicates [log_10_ copies/μl] ÷ average of replicates [log_10_ copies/μl]). All graphs were created using GraphPad Prism v9.3.1 statistical analysis software (GraphPad, San Diego, CA).

## Results

### Analytical specificity of singleplex and multiplex qPCR/RT-qPCR assays for the detection of feline respiratory pathogens

The analytical specificity (inclusivity/exclusivity) of all singleplex and multiplex qPCR/RT-qPCR assays were first evaluated using a panel of reference viruses and bacteria associated with respiratory, systemic, and enteric diseases in cats, as well as different SARS-CoV-2 variants of concern (VOC). All assays used and developed in this study showed exclusive specificity for their respective targets and did not cross-react between each other under multiplex conditions (S1 Fig). The specificity of the *M*. *felis* (tuf) assay was confirmed by absence of amplification of *M*. *canis* and *M*. *cynos* DNA extracts. Additionally, none of the assays amplified nucleic acids extracted from other feline viruses, including feline coronavirus (FCoV), feline infectious peritonitis virus (FIPV), and feline panleukopenia virus (FPLV).

### Analytical sensitivity of singleplex and multiplex qPCR/RT-qPCR assays for the detection of feline respiratory pathogens

**Fig 1:**
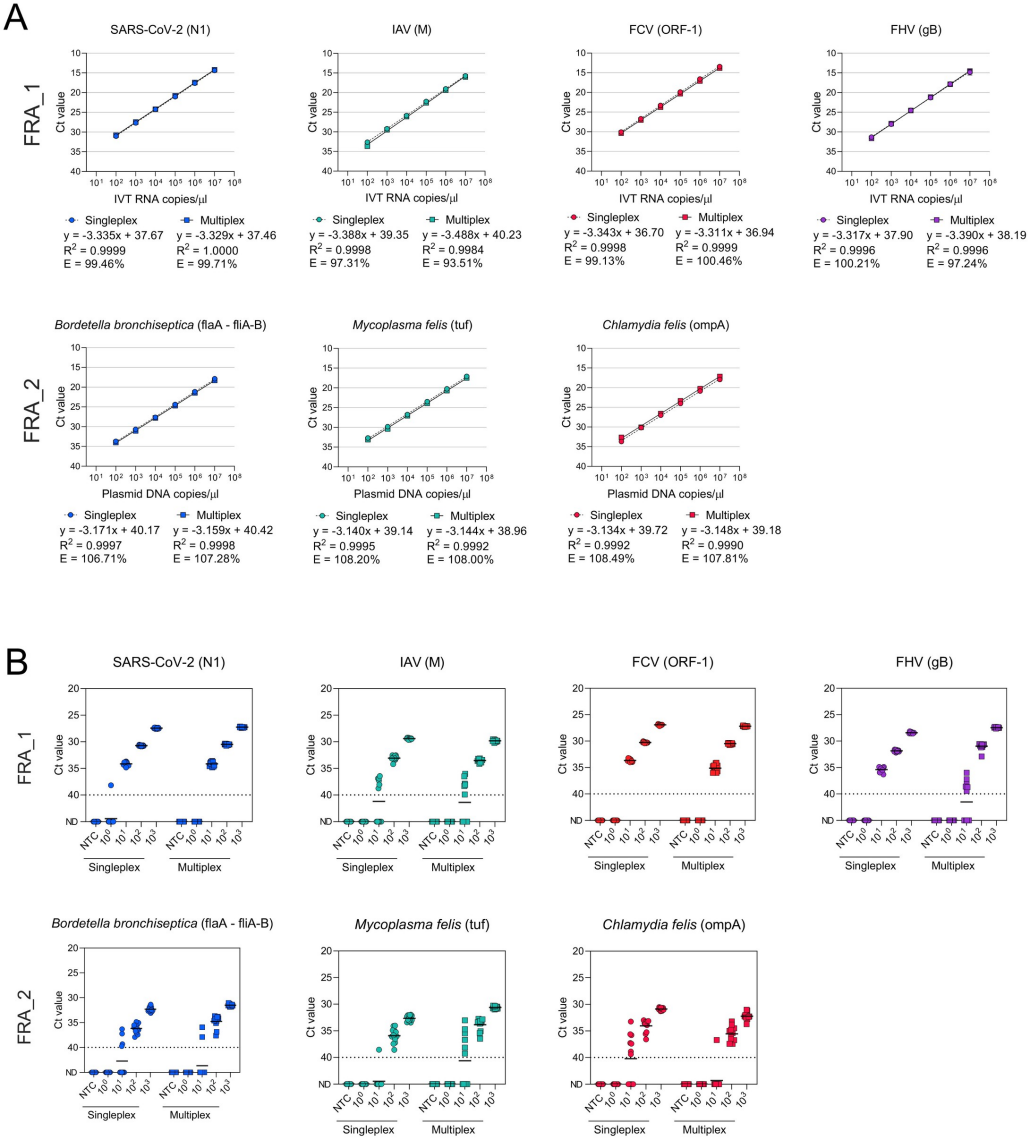
Analytical parameters of singleplex and multiplex qPCR/RT-qPCR assays for the detection of FRDC-associated pathogens and SARS-CoV-2. A) Comparison of analytical sensitivity of each singleplex and multiplex qPCR/RT-qPCR assays for the detection of pathogens associated with FRDC. B) Analytical sensitivity determination of singleplex and multiplex qPCR/RT-qPCR assays. Each assay was performed using 12 replicates ranging from 10^3^ to 10^0^ copies/μl of IVT DNA/RNA. Each circle and square indicate the Ct value of one replicate obtained by singleplex and multiplex amplification, respectively. Short solid lines indicate the median Ct value and dashed lines indicate the detection limit. Ct: Cycle threshold; IVT RNA: *in vitro* transcribed RNA; R^2^: linearity; E: Efficiency; ND: not detected; NTC: no template control.

The analytical sensitivity of all assays in singleplex and in multiplex format were determined using ten-fold serial dilutions (10^7^ copies/μl to 10^2^ copies/μl) of plasmid DNA/IVT RNA containing the target sequences. Linear standard curves were generated for each assay in singleplex and multiplex with a coefficient of linear regression (R^2^) ≥ 0.998 (Fig 1A, Table 3, S2 and S3 Figs). Amplification efficiency for each singleplex assay was between 97.31% and 108.49%. When tested in multiplex, a similar amplification efficiency was observed with values comprised between 93.51% and 107.81% (Table 3). The lower limit of detection (LOD_95%_) varied between 6 to 15 RNA/DNA copies/μl for each singleplex and multiplex assays (Fig 1B). A similar detection rate limit (100%) was calculated for each assay when used in both singleplex and multiplex conditions (10 to 100 copies/μl). Altogether, these results demonstrate the high analytical sensitivity of our panel of qPCR/RT-qPCR assays for the detection of feline respiratory pathogens, without loss of sensitivity when used in multiplex conditions.

**Table 3:** Analytical sensitivity of singleplex and multiplex qPCR/RT-qPCR assays for the detection of FRDC-associated pathogens and SARS-CoV-2.

Assay	Target	Parameter	Slope	Linearity (R^2^)	Efficiency (%)	LOD_95%_ (copies/μl)	Detection rate limit (copies/μl)	Ct cut-off
**FRA_1**	SARS-CoV-2 (N1 (ORF9a])	Singleplex	-3.335	0.9999	99.46	8	10	35
		Multiplex	-3.329	1.0000	99.71	15	100	35
	IAV (M)	Singleplex	-3.388	0.9998	97.31	15	100	34
		Multiplex	-3.488	0.9984	93.51	15	100	34
	FCV (ORF-1)	Singleplex	-3.343	0.9998	99.13	8	10	35
		Multiplex	-3.311	0.9999	100.46	8	10	37
	FHV-1 (gB [UL27])	Singleplex	-3.317	0.9996	100.21	6	10	37
		Multiplex	-3.390	0.9996	97.24	8	10	33
**FRA_2**	*B*. *bronchiseptica* (flaA—fliA-B)	Singleplex	-3.171	0.9997	106.71	9	100	39
		Multiplex	-3.159	0.9998	107.28	11	100	38
	*M*. *felis* (ompA)	Singleplex	-3.140	0.9995	108.20	15	100	40
		Multiplex	-3.151	0.9994	107.66	9	100	37
	*C*. *felis* (tuf)	Singleplex	-3.134	0.9992	108.49	15	100	40
		Multiplex	-3.148	0.9990	107.81	7	100	37

FRA: Feline respiratory assay; R^2^: Linearity; LOD_95%_: Limit of detection 95%; Ct: Cycle threshold.

### Repeatability and reproducibility of multiplex qPCR/RT-qPCR assays for detection of feline respiratory pathogens

The repeatability and reproducibility of both multiplex assays was measured by determining the intra-run and inter-run imprecision, respectively. A range of three concentrations; 10^5^ copies/μl (high target concentration), 10^4^ copies/μl (medium target concentration) and 10^3^ copies/μl (low target concentration), of plasmid DNA/IVT RNA was used to determine the coefficient of variability (CV) of each assay. For all assays the intra-run imprecision was < 1.5% at high target concentration, < 4% at medium target concentration and < 5% at low target concentration (Table 4). Similarly, the inter-run imprecision was < 3% at high target concentration, < 2.5% at medium target concentration and < 6% at low target concentration. While the CV increases with lower target concentrations, these data indicate that all multiplex assays have a high repeatability and reproducibility at high to low concentrations.

**Table 4:** Precision assessment of the feline respiratory assays 1 (FRA_1) and FRA_2.

Assay	Target	Intra-run variability CV (%)^#^			Inter-run variability CV (%)^#^		
		10^5^ copies/μl	10^4^ copies/μl	10^3^ copies/μl	10^5^ copies/μl	10^4^ copies/μl	10^3^ copies/μl
**FRA_1**	SARS-CoV-2 (N)	0.66	0.70	1.28	0.88	1.45	2.55
	IAV (M)	0.71	0.91	2.78	1.42	1.15	2.58
	FCV (ORF-1)	0.60	0.98	1.17	0.78	0.98	2.27
	FHV-1 (gB)	0.55	0.50	1.33	1.07	2.12	3.08
**FRA_2**	*B*. *bronchispetica* (flaA—fliA-B)	0.85	2.48	4.09	0.59	1.25	3.27
	*M*. *felis (tuf)*	1.27	3.53	4.88	2.86	2.09	5.41
	*C*. *felis* (ompA)	1.00	2.782	4.483	0.67	2.05	2.24

^#^CV (%): Coefficient of variation = (standard deviation of replicates [log_10_ copies/μl] ÷ Average of replicates [log_10_ copies/μl]) × 100

### Screening of clinical specimens collected from FRDC-suspected cats

**Fig 2:**
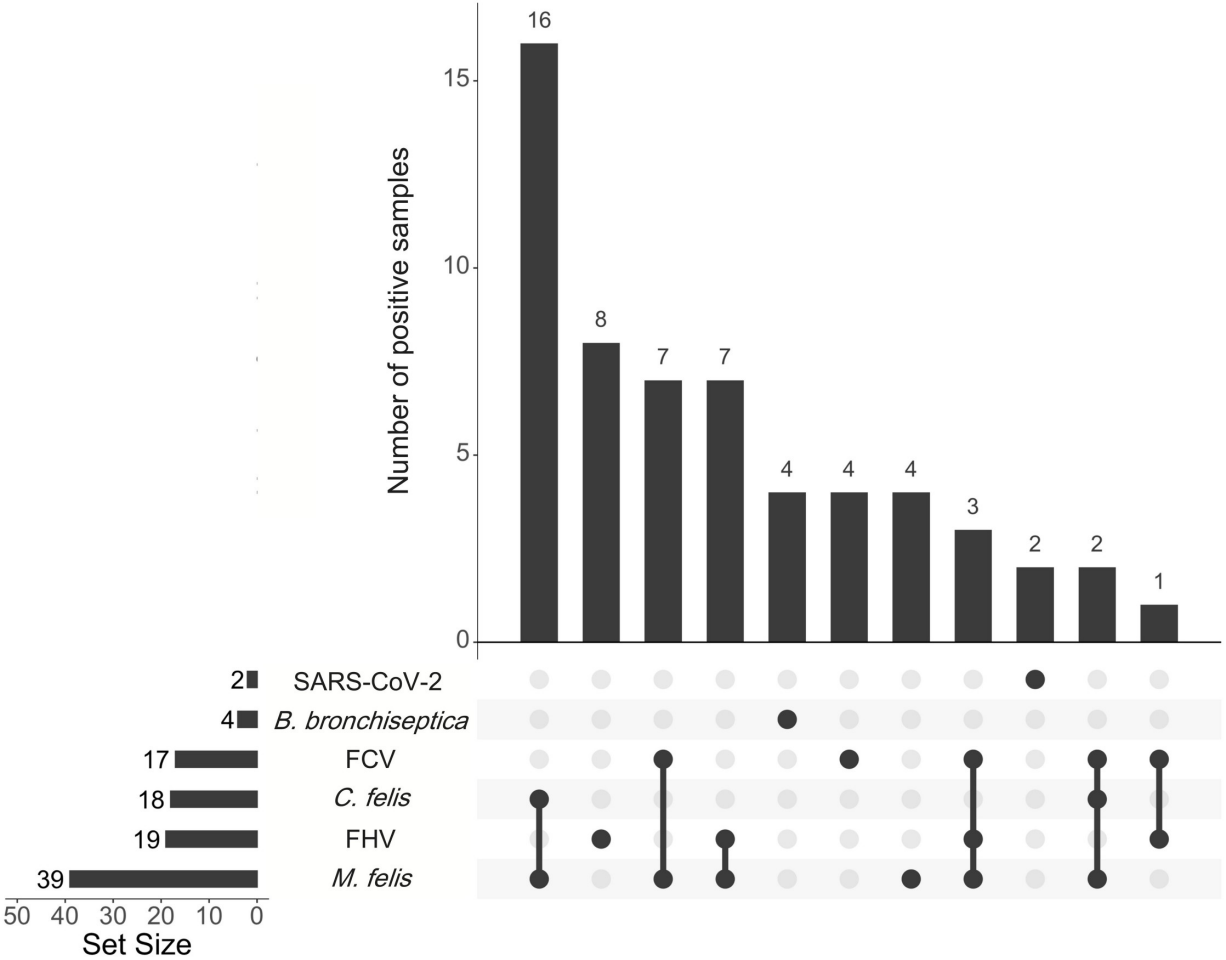
UpSet plot summarizing the number of feline respiratory pathogens and SARS-CoV-2 detected in felids using the newly developed multiplex qPCR/RT-qPCR panel. The number of samples with single agents detected or with multiple agents detected (co-infection) are shown as vertical bars. The bottom left horizontal bar graph labeled Set Size shows the total number of samples positives for each specific feline respiratory pathogen and SARS-CoV-2.

The panel of multiplex assays was used to test 63 clinical samples collected from domestic cats and exotic felids that displayed respiratory disease between 2020 and 2022 in Louisiana, USA. Among the 63 samples, 58 (92.1%) were positive for at least one of the seven pathogens screened. *M*. *felis* (61.9%) was the most commonly identified agent, followed by FHV-1 (30.2%), *C*. *felis* (28.7%) and FCV (27.0%) (Fig 2 and Table 5). *B*. *bronchiseptica* and SARS-CoV-2 were detected in four (6.3%) and two (3.2%) samples, respectively. None of the samples were positive for IAV. Both SARS-CoV-2 positive samples were collected from two 6-year-old female African lions with cough in 2021 within the same zoo. Partial sequencing of the Spike protein gene (ORF 2) performed by the National Veterinary Services Laboratories, Ames, IA, indicated that both SARS-CoV-2 isolates were consistent with the Delta variant (clade B.1.617.2). The complete sequences were previously deposited in GISAID database under accession numbers EPI_ISL_9046672 and EPI_ISL_9046673.

**Table 5:** Detection rate of FRDC-associated pathogens in the clinical specimens used to evaluate the FRA_ and FRA_2 assays.

Pathogens	No. of positives (n = 58/63)*	% positive (92.1%)*
SARS-CoV-2	2	3.2
IAV	0	0
FCV	17	27.0
FHV-1	19	30.2
*B*. *bronchiseptica*	4	6.3
*M*. *felis*	39	61.9
*C*. *felis*	18	28.7

*Positive samples for at least one pathogen

Single infections and co-infections were observed in 22 (34.9%) and 36 (57.1%) samples, respectively (Fig 2 and Table 6), demonstrating that infections with more than one FRDC- associated pathogen can occur more often. Except for the co-infection of FHV-1 with FCV in one sample, all other co-infected samples consisted of a combination of *M*. *felis* with FHV, FCV or *C*. *felis* (Fig 2 and Table 6). Co-infection of *M*. *felis* with *C*. *felis* was also commonly observed in the clinical specimens tested (16/36; 44.5%).

**Table 6:** Detection rate of single agent infections and co-infections associated with FRDC in the clinical specimens evaluated with FRA_1 and FRA_2 assays.

Pathogens	No. of Positive samples	% of Positive samples
***Respiratory infections associated with one pathogen***	***22/63*** *	**34.9**
SARS-CoV-2	2	3.2
FCV	4	6.3
*B*. *bronchiseptica*	4	6.3
*M*. *felis*	4	6.3
FHV-1	8	12.7
***Respiratory infections associated with two pathogens***	***31/63*** *	**49.2**
FHV-1 + FCV	1	1.6
FHV-1 + *M*. *felis*	7	11.1
FCV *+ M*. *felis*	7	11.1
*C*. *felis* + *M*. *felis*	16	25.4
***Respiratory infections associated with three pathogens***	***5/63*** *	**7.9**
FCV + *C*. *felis* + *M*. *felis*	2	3.2
FHV-1 + FCV + *M*. *felis*	3	4.8

*5/63 (7.9%) specimens were negative for the pathogens tested.

## Discussion

The most common pathogens reported to induce feline respiratory disease include FCV, FHV-1, *B*. *bronchiseptica*, *M*. *felis* and *C*. *felis* [3, 8]. The emergence of new pathogens (e.g. IAV and SARS-CoV-2) and the continuous circulation of common etiological agents in the feline population has made feline FRDC more complex and challenging its clinical diagnosis [14]. Additionally, co-infections by two or more viral and/or bacterial pathogens is commonly observed in cats suffering from FRDC, making the treatment challenging [74, 75]. The traditional methods for infectious agent identification, such as bacterial culture, viral isolation and conventional PCR are time consuming, have low sensitivity and are not suitable for easy identification of co-infections. Multiplex qPCR/RT-qPCR is a rapid and sensitive technique now commonly used in veterinary diagnostic laboratories [56–59]. However, there were no multiplex qPCR/RT-qPCR assays available for detection of infectious agents associated with FRDC. To overcome this, we developed a panel of two multiplex qPCR/RT-qPCR for the detection of the most prevalent feline respiratory pathogens as well as the detection of two emerging viruses of cats, IAV and SARS-CoV-2.

In this study two multiplex qPCR/RT-qPCR assays were developed, namely FRA_1 and FRA_2, for the detection of viruses (i.e., FCV, FHV-1, IAV and SARS-CoV-2) and bacteria (i.e., *B*. *bronchiseptica*, *M*. *felis* and *C*. *felis*), respectively. These multiplex assays were designed using a combination of well-established qPCR/RT-qPCR assays [63–68] and adding two new primers/probe combinations for the detection of FCV and FHV-1. These new primer/probe sets were developed targeting the highly conserved ORF1 and gB of FCV and FHV-1, respectively [76]. Both assays, as well as previously published assays showed high specificity for their targets alone and in combination, confirming the absence of non-specific amplification. Analytical sensitivity of each multiplex qPCR/RT-qPCR assays were evaluated in this study and compared to singleplex assays. The nearly perfect linearity (R^2^ > 0.998) and high amplification efficiency (> 93%) denotes the overall excellent analytical performance of each assay. In addition, no difference in analytical performance was observed between singleplex and multiplex formats of the assays. Detection of low genomic copy numbers is critical to assay’s sensitivity. Here, with a LOD_95%_ ≤ 15 copies/μl, a high analytical sensitivity was observed for all our primers and probe sets when multiplexed. The LOD_95%_ determined here showed a three to four log_10_ improvement compared to the recently published multiplex conventional PCR assays for the detection of FHV-1, FCV, IAV and *C*. *felis* (LOD were 1 × 10^4^ to 1 × 10^5^ copies/μL) [77]. Measuring both intra-run and inter-run assay variability is important to assess the assay’s quality and reproducibility. Here, a low intra-run and inter-run assay variabilities were observed for all assays when run in multiplex. This indicated that the qPCR/RT-qPCR assays are robust and consistent, providing confidence in the results obtained in this study. Overall, excellent analytical parameters were determined for both FRA_1 and FRA_2 multiplex assays.

It is worth noting that a single nucleotide degeneracy was introduced to the forward and reverse primers of the IAV assay, respectively, in order to empirically guarantee annealing to IAV strains derived from cats (n = 37) and retrieved from the Influenza Research Database. While it has been demonstrated that >4 degenerate nucleotides can lead to amplification bias and a reduction of the sensitivity (10-fold) [78–80], the introduction of one degenerate nucleotide per primer in this case did not seemingly affected the sensitivity of this or other targets combined (LOD_95%_ for IAV = 15 copies/μl in singleplex and multiplex conditions). The absence of IAV in any of the samples collected during the period of this study could be associated with the limited number of available samples during the study period as well as to the epidemiological occurrence of IAV in cats, which typically occur within distinct temporal and geographic locations. Hence, IAV infection in cats is relatively rare [16]. but their impact in cat populations and potential public health impact [15, 16], makes its diagnosis highly relevant.

To our knowledge, this study is the first reporting on the development of a complete panel of multiplex qPCR/RT-qPCR for the detection of feline respiratory pathogens along with SARS-CoV-2. Our panel was then used to evaluate clinical specimens collected from FRDC-suspected cats in different shelters, veterinary practices, and a zoo in Baton Rouge and the surrounding area between 2020 and 2022. As only a small number of samples were tested in this study, no conclusions can be made concerning the prevalence of the feline respiratory pathogens in this area. Nevertheless, a high rate of co-infections (57.1%) was detected in the samples collected here, supporting the need for simultaneous detection of multiple pathogens involved in FRDC. The most common infectious agent detected in this study was *M*. *felis* (61.9%), as consistently reported [12, 74, 81, 82]. A meta-analysis demonstrated that *M*. *felis* and FRDC were significantly associated [83], suggesting that *M*. *felis* may act as the initial pathogen that may predispose the cats to other viral and bacterial pathogens. However, the hypothesis that this bacterium may overgrow following damage as a consequence of FRDC needs to be considered, especially, because it was detected in almost all co-infected samples (35/36; 97.2%). However, further studies are still needed to determine the role of *M*. *felis* in feline FRDC. FCV, FHV-1 and *C*. *felis* were detected in approximately 30% of the tested samples, which is consistent with previous reports [8, 12, 74, 75, 81]. The detection of SARS-CoV-2 Delta VOC (B.1.617.2) in nasal swabs of two lions from the same premise by the CDC-developed RT-qPCR test [65] confirm that it can be multiplexed with other qPCR/RT-qPCR assays, as previously demonstrated [61, 62]. The use of SARS-CoV-2-positive nasal swabs from lions in this study was for the sole purpose of evaluating the performance of the panels developed. While domestic cats are susceptible to natural and experimental SARS-CoV-2 infections [39–49], SARS-CoV-2 has not, at least yet, established as a relevant pathogen in cats responsible for FRDC. However, screening for SARS-CoV-2 in cats with FRDC is relevant from a public health standpoint.

While the assays developed here demonstrated optimal analytical performance, one of the main limitations of this study is the limited number of available samples to perform thorough clinical performance evaluation. Thus, continued evaluation of clinical specimens to assess the assay’s performance in the field is warranted. In conclusion, this study highlights the strength of our new qPCR/RT-qPCR panel for the detection of SARS-CoV-2, IAV and the most important FRDC-associated pathogens. Therefore, this panel is suitable for routine diagnostics and rapid identification of pathogens associated with feline respiratory disease outbreaks in catteries, shelters, and pet shops where they house a large number of cats.

## Supporting information

S1 Fig Assessment of the specificity of each qPCR/RT-qPCR assay using reference viral and bacterial DNA and RNA. Each column corresponds to one specific qPCR/RT-qPCR assay and each row corresponds to one specific reference strain of virus or bacteria. Specificity was assessed for each assay in singleplex and in multiplex formats. White cases correspond to the absence of detection while black cases correspond to DNA/RNA amplification in both singleplex and multiplex assays.
(TIF)

S2 Fig Amplification curves generated under singleplex and multiplex conditions for a ten-fold serial dilution series of each target included in the FRA_1 assay. Each dilution was performed using three replicates ranging from 10^7^ to 10^1^ *IVT* RNA copies/μl. The X-axis represents the cycle number and the Y-axis represents the delta Rn value.
(TIF)

S3 Fig Amplification curves generated under singleplex and multiplex conditions for a ten-fold serial dilution series of each target included in the FRA_2 assay. Each dilution was performed using three replicates ranging from 10^7^ to 10^1^ plasmid DNA copies/μl. The X-axis represents the cycle number, and the Y-axis represents the delta Rn value.
(TIF)

S1 Table Origin and type of samples collected from URTD-suspected cats during this study. Thirty-nine nasal swabs and 24 pharyngeal swabs were collected from 39 felines from 2020 and 2022.
(DOCX)

S1 File Original data used to determine the analytical specificity, sensitivity, repeatability, and reproducibility of FRA_1 and FRA_2 assays. (PDF)
