# Supplementary figures and images for: Development and validation of multiplex one-step qPCR/RT-qPCR assays for simultaneous detection of SARS-CoV-2 and pathogens associated with feline respiratory disease complex
Source: PLoS One. 2024 Mar 22;19(3):e0297796. doi: 10.1371/journal.pone.0297796 (PMC10959388; doi:10.1371/journal.pone.0297796)

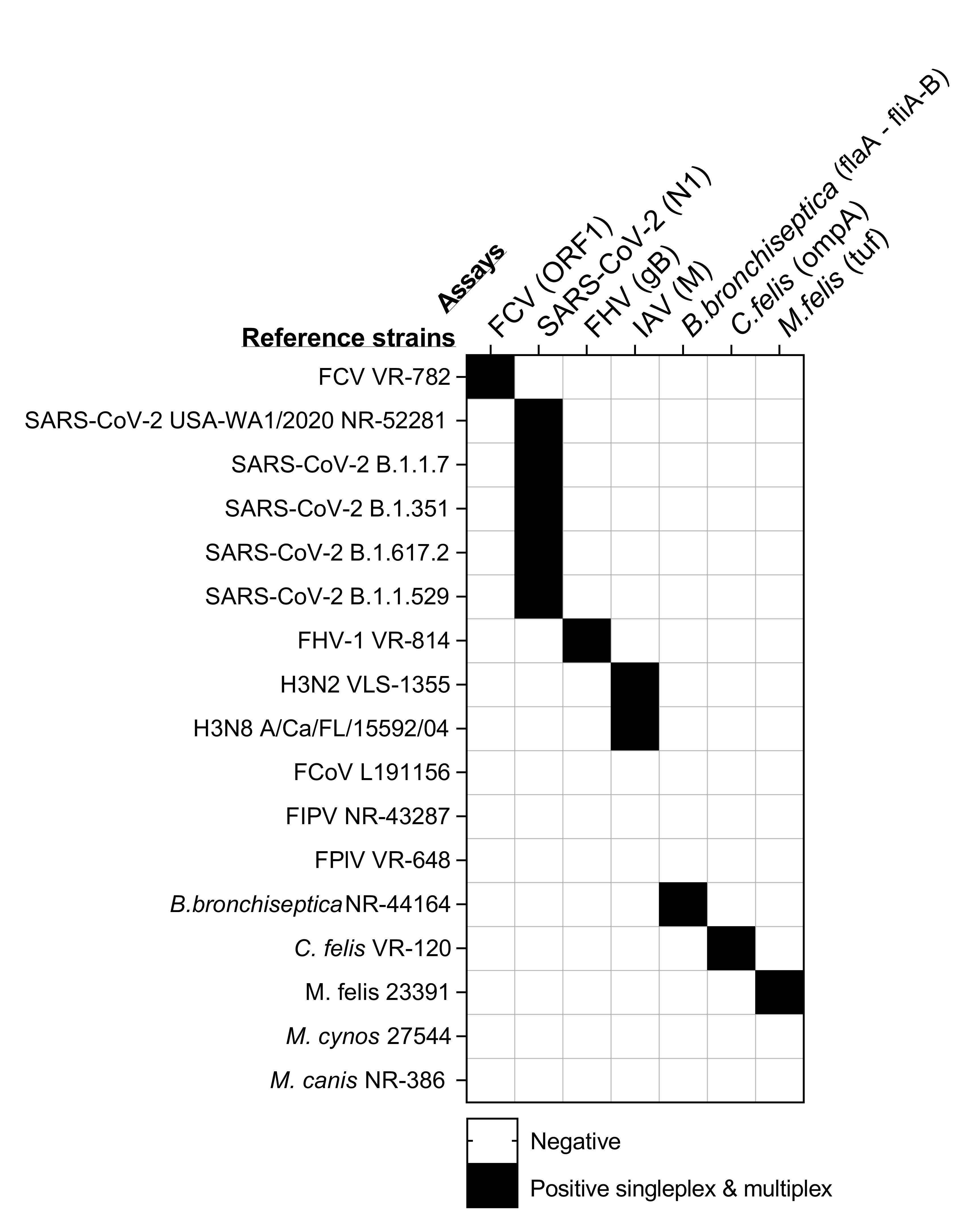

Supplement: S1 Fig — Each column corresponds to one specific qPCR/RT-qPCR assay and each row corresponds to one specific reference strain of virus or bacteria. Specificity was assessed for each assay in singleplex and in multiplex formats. White cases correspond to the absence of detection while black cases correspond to DNA/RNA amplification in both singleplex and multiplex assays. (TIF) [file pone.0297796.s001.tif]

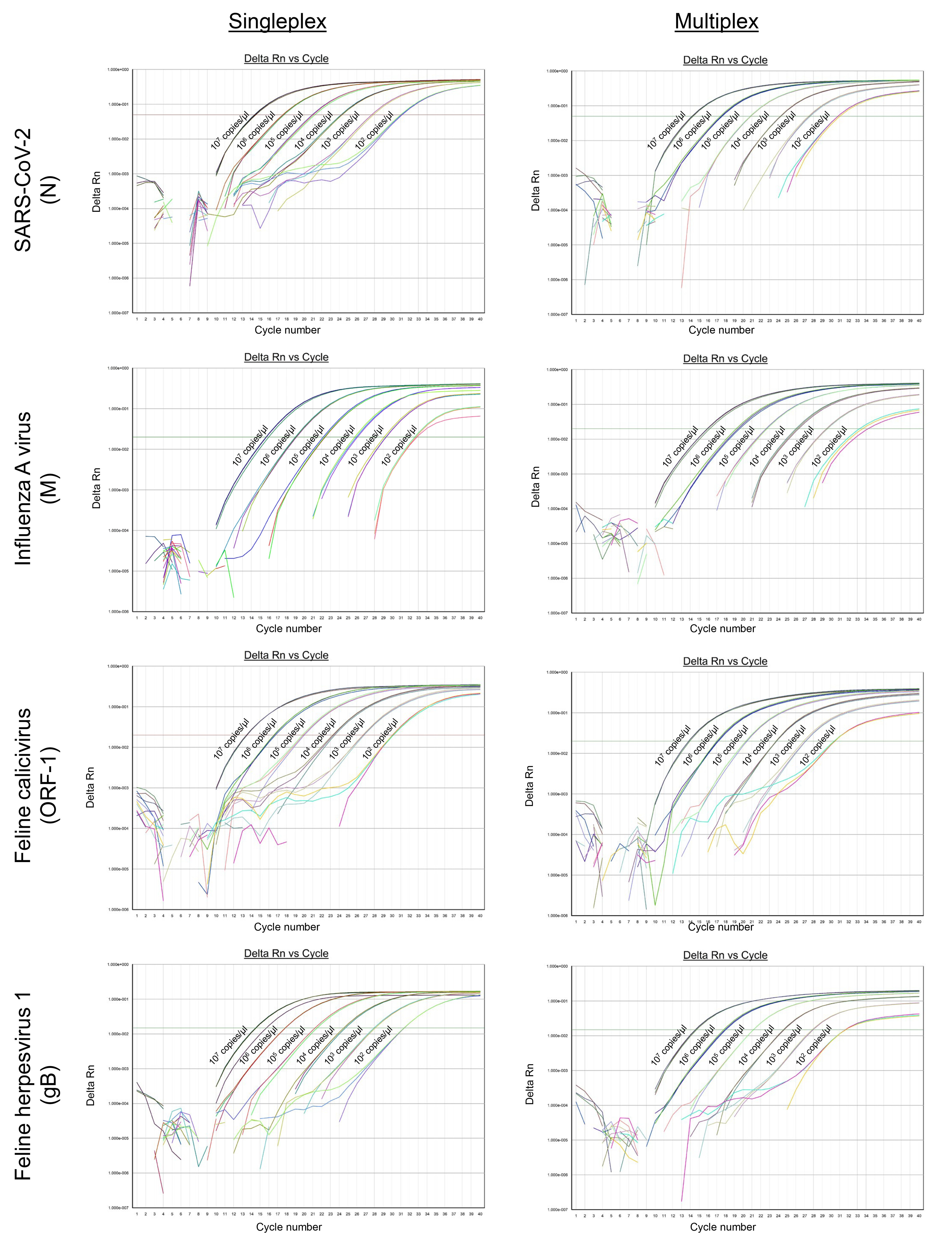

Supplement: S2 Fig — Each dilution was performed using three replicates ranging from 107 to 101 IVT RNA copies/μl. The X-axis represents the cycle number and the Y-axis represents the delta Rn value. (TIF) [file pone.0297796.s002.tif]

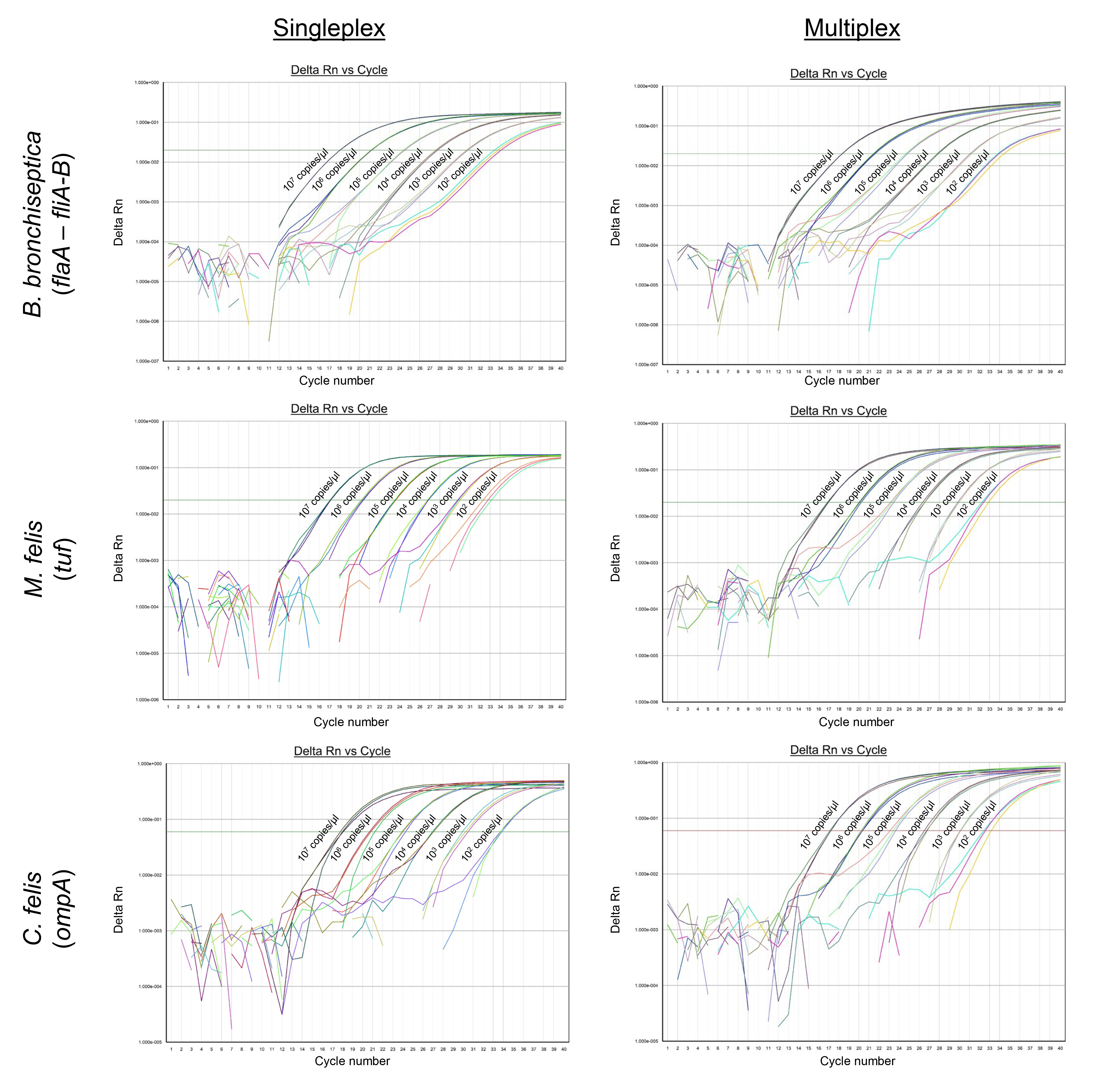

Supplement: S3 Fig — Each dilution was performed using three replicates ranging from 107 to 101 plasmid DNA copies/μl. The X-axis represents the cycle number, and the Y-axis represents the delta Rn value. (TIF) [file pone.0297796.s003.tif]
