# Supplementary material for: Development and validation of multiplex one-step qPCR/RT-qPCR assays for simultaneous detection of SARS-CoV-2 and pathogens associated with feline respiratory disease complex
Source: PLoS One. 2024 Mar 22;19(3):e0297796. doi: 10.1371/journal.pone.0297796 (PMC10959388; doi:10.1371/journal.pone.0297796)
Supplement: S1 Table — Thirty-nine nasal swabs and 24 pharyngeal swabs were collected from 39 felines from 2020 and 2022. (DOCX) [file pone.0297796.s004.docx]

**S1 Table:** Origin and type of samples collected from URTD-suspected cats during this study. Thirty-nine nasal swabs and 24 pharyngeal swabs were collected from 39 felines from 2020 and 2022.

| **Location** | **Felines #** | **Nasal swabs**  **(n=39)** | **Pharyngeal swabs**  **(n=24)** |
| --- | --- | --- | --- |
| Veterinary Practices and Zoological Gardens | 1 | X |  |
|  | 2 | X |  |
|  | 3 | X |  |
|  | 4 | X |  |
|  | 5 | X |  |
|  | 6 | X |  |
|  | 7 | X |  |
| Shelter 1 | 8 | X | X |
|  | 9 | X | X |
|  | 10 | X | X |
| Shelter 2 | 11 | X | X |
|  | 12 | X | X |
|  | 13 | X | X |
|  | 14 | X | X |
|  | 15 | X | X |
|  | 16 | X |  |
|  | 17 | X |  |
|  | 18 | X |  |
|  | 19 | X |  |
|  | 20 | X |  |
|  | 21 | X |  |
|  | 22 | X |  |
|  | 23 | X |  |
|  | 24 | X | X |
|  | 25 | X | X |
|  | 26 | X | X |
|  | 27 | X | X |
| Shelter 3 | 28 | X | X |
|  | 29 | X | X |
|  | 30 | X | X |
|  | 31 | X | X |
| Shelter 4 | 32 | X | X |
|  | 33 | X | X |
|  | 34 | X | X |
|  | 35 | X | X |
|  | 36 | X | X |
|  | 37 | X | X |
|  | 38 | X | X |
|  | 39 | X | X |
