# Supplementary material for: Development and validation of multiplex one-step qPCR/RT-qPCR assays for simultaneous detection of SARS-CoV-2 and pathogens associated with feline respiratory disease complex
Source: PLoS One. 2024 Mar 22;19(3):e0297796. doi: 10.1371/journal.pone.0297796 (PMC10959388; doi:10.1371/journal.pone.0297796)
Supplement: S1 File — (PDF) [file pone.0297796.s005.pdf]

FRA_1 assay

Standard curves

Multiplex												
**Dye**	**ABY**			**Cy5**			**FAM**			**VIC**		
**Target (copies/µL)**	**FCV**			**FHV**			**SARS-CoV-2**			**IAV**		
**1.00E+02**	30.1179	30.026	30.0495	31.3249	31.3327	31.3082	31.04	31.12	30.95	32.4804	32.7722	32.6143
**1.00E+03**	26.7761	26.6793	26.5999	27.9399	28.0666	28.0668	27.61	27.62	27.82	29.2269	29.2614	29.0599
**1.00E+04**	23.4183	23.2811	23.1997	24.6359	24.4505	24.4518	24.16	24.25	24.27	25.8795	26.0296	25.6692
**1.00E+05**	20.0169	19.8947	19.7477	21.2041	21.2095	21.4253	20.89	21.01	21.18	22.1252	22.3099	22.2612
**1.00E+06**	16.679	16.5346	16.4926	17.8384	17.8469	17.8142	17.65	17.54	17.65	18.9698	19.087	19.0312
**1.00E+07**	13.3801	13.4484	13.4202	14.4242	15.6671	14.4948	14.3	14.37	14.45	15.5866	15.6411	15.9422

Singleplex												
**Dye**	**ABY**			**Cy5**			**FAM**			**VIC**		
**Target (copies/µL)**	**FCV**			**FHV**			**SARS-CoV-2**			**IAV**		
**1.00E+02**	30.3033	30.3795	30.3616	31.59	31.5857	31.645	30.71	30.72	30.97	33.2054	34.1841	33.5299
**1.00E+03**	27.1061	27.0526	26.84	27.9185	27.9389	27.8127	27.56	27.45	27.36	29.531	29.4015	29.4785
**1.00E+04**	23.754	23.7092	23.6812	24.5091	24.5989	24.5294	24.18	24.22	24.18	25.9882	26.2387	26.104
**1.00E+05**	20.3765	20.2837	20.2512	21.1355	21.1471	21.1347	20.82	20.8	20.76	22.5235	22.6178	22.6659
**1.00E+06**	17.1479	17.0798	16.9894	18.0234	17.8966	17.7378	17.54	17.45	17.34	19.5309	19.3585	19.1713
**1.00E+07**	13.7686	13.9034	13.7511	14.5419	14.6456	14.4965	14.18	14.25	14.13	15.9417	16.162	15.8797

LOD95%,Detection rate limit & Ct cut-off

Multiplex																								
**Dye**	**ABY**						**Cy5**						**FAM**						**VIC**					
**Target**	**FCV**						**FHV**						**SARS-CoV-2**						**IAV**					
**replicat #**	**1.00E+05**	**1.00E+04**	**1.00E+03**	**1.00E+02**	**1.00E+01**	**1.00E+00**	**1.00E+05**	**1.00E+04**	**1.00E+03**	**1.00E+02**	**1.00E+01**	**1.00E+00**	**1.00E+05**	**1.00E+04**	**1.00E+03**	**1.00E+02**	**1.00E+01**	**1.00E+00**	**1.00E+05**	**1.00E+04**	**1.00E+03**	**1.00E+02**	**1.00E+01**	**1.00E+00**
**1**	20.287	23.9356	27.3226	30.6025	35.2674	Undetermi	21.4587	24.1592	27.52	31.1246	Undetermi	Undetermi	21.0168	24.0155	27.3922	30.5768	34.0462	Undetermi	22.1661	26.1979	29.7875	33.5668	Undetermi	Undetermi
**2**	20.1963	23.8589	27.1369	30.3535	34.7588	Undetermi	21.4546	24.1129	27.4713	30.8492	Undetermi	Undetermi	20.9466	23.9946	27.209	30.4892	34.5622	Undetermi	22.2039	26.1723	29.7313	33.3974	Undetermi	Undetermi
**3**	20.0707	23.7726	27.1934	30.3262	34.8158	Undetermi	21.3018	24.1682	27.5074	30.7661	Undetermi	Undetermi	20.7821	23.923	27.2335	30.4373	34.0591	Undetermi	22.0422	26.3452	29.8493	33.576	Undetermi	Undetermi
**4**	20.1813	23.6907	27.1546	30.5893	34.1536	Undetermi	21.4156	24.0954	27.4714	30.6001	Undetermi	Undetermi	20.9303	23.8479	27.2476	30.469	33.6166	Undetermi	22.2025	26.3268	30.0027	33.3955	39.8837	Undetermi
**5**	20.2136	23.6384	27.1245	30.3363	35.3333	Undetermi	21.5381	24.064	27.5761	30.7009	Undetermi	Undetermi	21.0022	23.8338	27.1999	30.4897	33.8007	Undetermi	22.2747	26.2572	29.5396	33.1817	Undetermi	Undetermi
**6**	20.1356	23.7386	27.1974	30.3986	35.8701	Undetermi	21.4573	24.091	27.3915	30.5666	37.2018	Undetermi	20.9309	23.9002	27.2583	30.3468	34.007	Undetermi	22.1783	26.2668	29.5273	33.1346	37.8723	Undetermi
**7**	20.0846	23.729	27.1197	30.3497	34.6324	Undetermi	21.4097	24.0685	27.3515	30.6902	35.9849	Undetermi	20.931	23.872	27.2408	30.4933	33.7004	Undetermi	22.2854	26.2603	29.8828	33.3926	36.0016	Undetermi
**8**	20.127	23.691	27.2616	30.6653	35.723	Undetermi	21.3664	24.0558	27.6121	30.5664	Undetermi	Undetermi	20.9617	23.8613	27.3563	30.4662	34.8042	Undetermi	22.2387	26.2574	30.2001	33.4077	38.2842	Undetermi
**9**	20.1301	23.7658	27.0365	30.7455	34.0793	Undetermi	21.4352	24.099	27.3001	31.1898	39.4253	Undetermi	20.9624	23.8994	27.1423	30.7177	34.0725	Undetermi	22.3206	26.3436	29.6183	34.1237	36.4801	Undetermi
**10**	20.2402	23.7317	27.1698	30.4792	36.0116	Undetermi	21.5088	23.9971	27.3916	30.9028	38.1474	Undetermi	21.0503	23.8427	27.2201	30.3701	34.6295	Undetermi	22.2603	26.0483	29.915	33.8775	38.063	Undetermi
**11**	20.2666	23.7658	27.2245	30.362	34.6179	Undetermi	21.5676	23.9877	27.3447	30.6732	38.5987	Undetermi	21.0396	23.8404	27.2575	30.4196	33.7735	Undetermi	22.4639	26.0809	29.7867	33.2165	Undetermi	Undetermi
**12**	20.3223	23.9499	27.2326	30.4911	36.0053	Undetermi	21.4985	24.0762	27.3879	32.8964	38.6945	Undetermi	21.0681	23.9352	27.2007	30.4684	34.396	Undetermi	22.1722	26.1903	29.8675	33.8728	Undetermi	Undetermi
**Average**	**20.19**	**23.77**	**27.18**	**30.47**	**35.11**	**N.A.**	**21.45**	**24.08**	**27.44**	**30.96**	**38.01**	**N.A.**	**20.97**	**23.90**	**27.25**	**30.48**	**34.12**	**N.A.**	**22.23**	**26.23**	**29.81**	**33.51**	**37.76**	**N.A.**
**STDEV**	**0.08**	**0.10**	**0.08**	**0.14**	**0.69**	**N.A.**	**0.07**	**0.05**	**0.10**	**0.64**	**1.23**	**N.A.**	**0.08**	**0.06**	**0.07**	**0.10**	**0.39**	**N.A.**	**0.10**	**0.10**	**0.19**	**0.31**	**1.39**	**N.A.**
**Ct cut-off**	**20**	**24**	**27**	**31**	**37**	**N.A.**	**22**	**24**	**28**	**33**	**42**	**N.A.**	**21**	**24**	**27**	**31**	**35**	**N.A.**	**23**	**27**	**30**	**34**	**42**	**N.A.**
**LOD95%**	**8**						**15**						**8**						**15**					

Singleplex																				
**Dye**	**ABY**					**Cy5**					**FAM**					**VIC**				
**Target**	**FCV**					**FHV**					**SARS-CoV-2**					**IAV**				
**replicat #**	**1.00E+04**	**1.00E+03**	**1.00E+02**	**1.00E+01**	**1.00E+00**	**1.00E+04**	**1.00E+03**	**1.00E+02**	**1.00E+01**	**1.00E+00**	**1.00E+04**	**1.00E+03**	**1.00E+02**	**1.00E+01**	**1.00E+00**	**1.00E+04**	**1.00E+03**	**1.00E+02**	**1.00E+01**	**1.00E+00**
**1**	23.6798	26.909	30.3395	33.6073	Undeterm	25.1596	28.2221	31.6701	35.2977	Undetermi	24.2418	27.5652	30.8563	33.6928	Undetermi	26.0759	29.2175	33.3761	36.8887	Undetermi
**2**	23.4892	26.9558	30.2378	33.4786	Undeterm	24.8024	34.7836	31.6522	35.6363	Undetermi	24.1778	27.5587	30.7642	34.0306	Undetermi	26.0772	29.2192	32.5278	Undetermi	Undetermi
**3**	23.4915	26.8464	30.2432	33.7594	Undeterm	24.7759	28.4162	31.9741	36.2993	Undetermi	23.9733	27.5879	30.79	34.287	Undetermi	26.0189	29.5927	32.529	37.2846	Undetermi
**4**	23.4227	26.7452	30.0185	33.4417	Undeterm	24.7935	28.4442	32.1757	35.2437	Undetermi	24.0064	27.4579	30.5415	33.7995	Undetermi	26.0547	29.2605	33.4348	36.8764	Undetermi
**5**	23.4743	26.9019	30.1595	33.9474	Undeterm	24.7953	28.5351	31.9702	35.4343	Undetermi	23.9138	27.5004	30.6028	34.4449	Undetermi	25.925	29.3165	32.975	Undetermi	Undetermi
**6**	23.5649	26.8566	30.2626	33.9641	Undeterm	24.9048	28.2685	31.5707	34.8742	Undetermi	23.9872	27.3517	30.7029	34.1029	Undetermi	25.666	29.5197	32.8378	Undetermi	Undetermi
**7**	23.5669	26.863	30.2941	33.4123	Undeterm	25.0105	28.4065	31.7533	34.8816	Undetermi	24.0108	27.3248	30.6226	33.7809	38.1562	26.0896	29.411	32.598	38.7482	Undetermi
**8**	23.514	26.9128	30.2373	33.265	Undeterm	24.8707	28.528	31.6363	35.0448	Undetermi	23.9817	27.3364	30.8552	34.0361	Undetermi	26.011	29.3876	32.9648	Undetermi	Undetermi
**9**	23.4406	26.8889	30.1444	33.1975	Undeterm	25.1217	28.548	31.82	35.9208	Undetermi	24.1452	27.3187	30.666	34.3227	Undetermi	25.9736	29.3873	33.2328	Undetermi	Undetermi
**10**	23.5873	26.9189	30.1795	33.9459	Undeterm	25.0621	28.1286	31.9721	35.1994	Undetermi	24.032	27.3885	30.7094	33.8504	Undetermi	26.0713	29.6322	34.1842	36.4187	Undetermi
**11**	23.5894	27.0079	30.3827	34.0138	Undeterm	25.0253	28.3355	31.9734	35.4775	Undetermi	24.1047	27.34	30.6102	34.473	Undetermi	26.0566	29.3405	33.0873	38.0276	Undetermi
**12**	23.7224	27.1436	30.4092	33.7327	Undeterm	24.9514	28.5601	31.8909	35.4011	Undetermi	24.2065	27.4144	30.804	34.8145	Undetermi	26.1432	29.4738	33.1197	Undetermi	Undetermi
**Average**	**23.55**	**26.91**	**30.24**	**33.65**	**N.A.**	**24.94**	**28.93**	**31.84**	**35.39**	**N.A.**	**24.07**	**27.43**	**30.71**	**34.14**	**38.16**	**26.01**	**29.40**	**33.07**	**37.37**	**N.A.**
**STDEV**	**0.09**	**0.10**	**0.11**	**0.29**	**N.A.**	**0.14**	**1.85**	**0.18**	**0.41**	**N.A.**	**0.11**	**0.10**	**0.10**	**0.34**	**N.A.**	**0.12**	**0.14**	**0.47**	**0.86**	**N.A.**
**Ct cut-off**	**24**	**27**	**31**	**35**	**N.A.**	**25**	**34**	**32**	**37**	**N.A.**	**24**	**28**	**31**	**35**	**N.A.**	**26**	**30**	**34**	**40**	**N.A.**
**LOD95%**	**8**					**8**					**6**					**15**				

Intra-run variability

**a**	-3.311			-3.39			-3.308			-3.488		
**b**	33.63			34.8			32.56			36.74		
**1**	4.03	2.93	1.90	3.94	3.14	2.15	3.49	2.58	1.56	4.18	3.02	1.99
**2**	4.06	2.95	1.96	3.94	3.15	2.16	3.51	2.59	1.62	4.17	3.03	2.01
**3**	4.10	2.98	1.94	3.98	3.14	2.15	3.56	2.61	1.61	4.21	2.98	1.98
**4**	4.06	3.00	1.96	3.95	3.16	2.16	3.52	2.63	1.61	4.17	2.99	1.93
**5**	4.05	3.02	1.96	3.91	3.17	2.13	3.49	2.64	1.62	4.15	3.01	2.06
**6**	4.08	2.99	1.94	3.94	3.16	2.19	3.52	2.62	1.60	4.17	3.00	2.07
**7**	4.09	2.99	1.97	3.95	3.17	2.20	3.52	2.63	1.61	4.14	3.00	1.97
**8**	4.08	3.00	1.92	3.96	3.17	2.12	3.51	2.63	1.57	4.16	3.01	1.87
**9**	4.08	2.98	1.99	3.94	3.16	2.21	3.51	2.62	1.64	4.13	2.98	2.04
**10**	4.04	2.99	1.95	3.92	3.19	2.19	3.48	2.64	1.61	4.15	3.07	1.96
**11**	4.04	2.98	1.93	3.90	3.19	2.20	3.48	2.64	1.60	4.09	3.06	1.99
**12**	4.02	2.92	1.93	3.92	3.16	2.19	3.47	2.61	1.62	4.18	3.02	1.97
**AVERAGE**	**4.06**	**2.98**	**1.95**	**3.94**	**3.16**	**2.17**	**3.50**	**2.62**	**1.61**	**4.16**	**3.01**	**1.99**
**STDEV**	**0.02**	**0.03**	**0.02**	**0.02**	**0.02**	**0.03**	**0.02**	**0.02**	**0.02**	**0.03**	**0.03**	**0.06**
**%CV**	**0.60**	**0.98**	**1.17**	**0.55**	**0.50**	**1.33**	**0.66**	**0.70**	**1.28**	**0.71**	**0.91**	**2.78**

Inter-run variability

**replicat**	**1.00E+05**	**1.00E+04**	**1.00E+03**	**1.00E+05**	**1.00E+04**	**1.00E+03**	**1.00E+05**	**1.00E+04**	**1.00E+03**	**1.00E+05**	**1.00E+04**	**1.00E+03**
**1**	20.38	23.75	27.11	21.14	24.51	27.92	20.82	24.18	27.56	22.52	25.99	29.53
**2**	20.28	23.71	27.05	21.15	24.6	27.94	20.8	24.22	27.45	22.62	26.24	29.4
**3**	20.25	23.68	26.84	21.14	24.53	27.81	20.76	24.18	27.36	22.67	26.1	29.48
**1**	4.00	2.98	1.97	4.03	3.04	2.03	3.55	2.53	1.51	4.08	3.08	2.07
**2**	4.03	3.00	1.99	4.03	3.01	2.02	3.56	2.52	1.54	4.05	3.01	2.10
**3**	4.04	3.01	2.05	4.03	3.03	2.06	3.57	2.53	1.57	4.03	3.05	2.08
**AVERAGE**	4.04	2.97	1.97	3.99	3.08	2.10	3.54	2.56	1.57	4.09	3.03	2.04
**STDEV**	0.0313829	0.0290222	0.048445	0.042632	0.0654452	0.064592	0.031169	0.0371518	0.040015	0.055865	0.034777	0.05264
**%CV**	**0.78**	**0.98**	**2.45**	**1.07**	**2.12**	**3.08**	**0.88**	**1.45**	**2.55**	**1.37**	**1.15**	**2.58**

FRA_2 assay

Standard curves

Multiplex

Dye	FAM			ABY			VIC		
Target (copies/µL	*B. bronchiseptica*			*C. felis*			*M. felis*		
**1.00E+02**	33.3597	34.0506	33.7113	33.7928	33.6334	33.6483	33.0819	32.6959	32.2194
**1.00E+03**	30.7328	30.6835	30.5749	29.9029	30.4174	30.1991	29.771	29.7001	29.9736
**1.00E+04**	27.6941	27.689	27.5289	26.92	27.0355	27.0385	26.8033	26.7661	26.6932
**1.00E+05**	24.4306	24.3833	24.327	24.2148	23.981	23.883	23.4505	23.5128	23.4545
**1.00E+06**	21.1536	21.1684	21.1341	21.1821	20.6355	20.7838	20.2486	20.3229	20.1449
**1.00E+07**	17.515	18.0547	18.0329	17.9898	18.0549	17.7396	17.1012	17.1033	17.059

Single-plex

Dye	FAM			ABY			VIC		
Target (copies/µL	*B. bronchiseptica*			*C. felis*			*M. felis*		
**1.00E+02**	33.7561	33.8686	34.3714	32.3522	32.4897	33.1156	32.85	32.99	33.43
**1.00E+03**	31.0498	31.0324	31.1507	30.087	30.059	30.047	30.36	30.35	30.46
**1.00E+04**	27.8491	27.7302	27.7464	26.672	26.4225	26.7141	27.14	26.94	27.1
**1.00E+05**	24.762	24.6219	24.6045	23.5755	23.075	23.3601	24.03	23.78	23.9
**1.00E+06**	21.5439	21.4355	21.3727	20.3064	20.297	20.1868	20.82	20.65	20.59
**1.00E+07**	18.2983	18.2826	18.2682	17.1757	17.0932	17.1736	17.51	17.46	17.49

LOD95%,Detection rate limit & Ct cut-off

Multiplex

	FAM						ABY						VIC					
	B. bronchiseptica						*C. felis*						*M. felis*					
**replicat #**	**1.00E+05**	**1.00E+04**	**1.00E+03**	**1.00E+02**	**1.00E+01**	**1.00E+00**	**1.00E+05**	**1.00E+04**	**1.00E+03**	**1.00E+02**	**1.00E+01**	**1.00E+00**	**1.00E+05**	**1.00E+04**	**1.00E+03**	**1.00E+02**	**1.00E+01**	**1.00E+00**
**1**	**24.6895**	27.9261	31.5557	37.6133	Undetermi	Undetermi	24.291	27.2508	31.2009	35.5471	38.8676	Undetermi	23.9994	26.949	30.808	35.0648	Undetermi	Undetermi
**2**	**24.8841**	27.501	31.217	35.106	Undetermi	Undetermi	24.2426	26.6939	30.6532	33.5705	37.26	Undetermi	24.0545	26.4283	30.2957	33.5539	38.1363	Undetermi
**3**	**24.4968**	27.9611	31.8141	34.1051	Undetermi	Undetermi	23.8372	27.3168	30.9315	33.535	Undetermi	Undetermi	23.68	26.9395	30.6998	33.159	Undetermi	Undetermi
**4**	**24.5403**	27.6041	31.5793	36.8406	Undetermi	Undetermi	24.0518	26.8156	30.8582	35.2326	Undetermi	Undetermi	23.7403	26.6559	30.7633	35.4104	Undetermi	Undetermi
**5**	**24.6828**	27.8625	31.8229	34.7107	Undetermi	Undetermi	24.2183	26.9405	31.2975	33.8984	Undetermi	Undetermi	24.0285	26.7912	30.9799	33.6374	Undetermi	Undetermi
**6**	**24.7471**	27.2995	31.7807	33.6709	Undetermi	Undetermi	24.1134	26.4686	31.1803	32.9735	33.2296	Undetermi	23.9947	26.2799	30.8545	32.7608	33.0268	Undetermi
**7**	**24.6415**	27.6527	31.4427	34.1107	Undetermi	Undetermi	24.0012	26.8972	30.5161	33.3171	Undetermi	Undetermi	23.9029	26.6674	30.4539	33.2386	39.2714	Undetermi
**8**	**24.5124**	27.3591	31.3091	34.3876	Undetermi	Undetermi	23.9238	26.4014	30.8524	33.6176	39.3626	Undetermi	23.7667	26.2828	30.6257	33.3731	Undetermi	Undetermi
**9**	**24.649**	27.6502	31.6078	34.61	Undetermi	Undetermi	23.7981	27.0518	31.0148	33.6954	37.3851	Undetermi	23.7646	26.6911	30.8002	33.3325	36.7002	Undetermi
**10**	**24.7058**	27.8764	31.3665	34.246	37.8834	Undetermi	24.0396	27.0564	30.6219	33.1914	35.7798	Undetermi	23.9151	26.7888	30.4373	33.1872	35.4628	Undetermi
**11**	**24.645**	27.9205	31.0367	33.7931	35.9166	Undetermi	24.2002	27.1118	30.624	33.1333	35.6211	Undetermi	23.9258	26.8307	30.271	32.8248	34.6987	Undetermi
**12**	**24.7762**	28.0511	31.378	33.9043	Undetermi	Undetermi	24.0413	27.5353	30.6364	36.573	Undetermi	Undetermi	23.9323	27.1376	30.3251	36.4637	Undetermi	Undetermi
**Average**	**24.66**	**27.72**	**31.49**	**34.76**	**36.90**	**#DIV/0!**	**24.06**	**26.96**	**30.87**	**34.02**	**36.79**	**#DIV/0!**	**23.89**	**26.70**	**30.61**	**33.83**	**36.22**	**#DIV/0!**
**STDEV**	**0.11**	**0.25**	**0.25**	**1.23**	**1.39**	**#DIV/0!**	**0.16**	**0.33**	**0.26**	**1.13**	**2.10**	**#DIV/0!**	**0.12**	**0.26**	**0.24**	**1.16**	**2.29**	**#DIV/0!**
**Ct cut-off**	**25**	**28**	**32**	**38**	**41**	**#DIV/0!**	**25**	**28**	**32**	**37**	**43**	**#DIV/0!**	**24**	**27**	**31**	**37**	**43**	**#DIV/0!**
**LOD95%**	**11**						**7**						**9**					

Single-plex

	FAM					ABY					VIC				
	B. bronchiseptica					*C. felis*					*M. felis*				
**replicat #**	**1.00E+04**	**1.00E+03**	**1.00E+02**	**1.00E+01**	**1.00E+00**	**1.00E+04**	**1.00E+03**	**1.00E+02**	**1.00E+01**	**1.00E+00**	**1.00E+04**	**1.00E+03**	**1.00E+02**	**1.00E+01**	**1.00E+00**
**1**	28.5081	31.8663	37.8994	39.299	Undetermi	28.6059	32.3042	37.405	Undetermi	Undetermi	28.7611	32.1436	34.9064	38.5339	Undetermir
**2**	28.372	31.6505	35.9713	Undetermi	Undetermi	28.7554	33.7011	35.3529	Undetermi	Undetermi	28.8949	33.4344	37.2247	Undetermi	Undetermir
**3**	28.3144	33.0836	37.4347	Undetermi	Undetermi	28.6825	31.1675	34.9914	Undetermi	Undetermi	29.0663	32.9811	36.076	Undetermi	Undetermir
**4**	28.1414	32.7673	35.494	Undetermi	Undetermi	28.1071	32.0886	33.2204	Undetermi	Undetermi	29.1744	32.9879	34.0923	Undetermi	Undetermir
**5**	28.1012	32.6678	35.0193	Undetermi	Undetermi	28.3587	32.278	35.4141	Undetermi	Undetermi	29.1048	33.3894	34.9079	Undetermi	Undetermir
**6**	28.3638	32.4033	36.9997	Undetermi	Undetermi	28.4061	32.2939	36.1778	36.7042	Undetermi	29.0392	32.0658	36.0197	Undetermi	Undetermir
**7**	28.2964	32.5496	34.8302	Undetermi	Undetermi	27.8638	32.0047	36.7217	Undetermi	Undetermi	29.3501	32.3181	35.2355	Undetermi	Undetermir
**8**	28.1589	31.8871	35.5259	Undetermi	Undetermi	28.2699	32.7606	34.481	Undetermi	Undetermi	28.9977	31.9468	36.5522	Undetermi	Undetermir
**9**	28.5147	32.32	35.5	36.3093	Undetermi	28.4584	31.0269	36.4804	Undetermi	Undetermi	28.9075	33.0798	38.5525	Undetermi	Undetermir
**10**	28.238	31.3455	36.4778	39.7575	Undetermi	28.4213	32.2679	34.7771	Undetermi	Undetermi	28.8619	32.4355	35.8298	Undetermi	Undetermir
**11**	28.3163	32.5647	36.3225	Undetermi	Undetermi	28.6505	32.483	37.4123	Undetermi	Undetermi	29.2009	32.8711	37.43	Undetermi	Undetermir
**12**	28.4724	32.4218	36.705	37.2097	Undetermi	28.0598	32.3634	33.8054	Undetermi	Undetermi	29.0849	32.0289	34.026	Undetermi	Undetermir
**Average**	**28.32**	**32.29**	**36.18**	**38.14**	**#DIV/0!**	**28.39**	**32.23**	**35.52**	**36.70**	**#DIV/0!**	**29.04**	**32.64**	**35.90**	**38.53**	**#DIV/0!**
**STDEV**	**0.14**	**0.51**	**0.96**	**1.65**	**#DIV/0!**	**0.27**	**0.69**	**1.35**	**#DIV/0!**	**#DIV/0!**	**0.16**	**0.54**	**1.38**	**#DIV/0!**	**#DIV/0!**
**Ct cut-off**	**29**	**34**	**39**	**43**	**#DIV/0!**	**29**	**34**	**40**	**#DIV/0!**	**#DIV/0!**	**30**	**34**	**40**	**#DIV/0!**	**#DIV/0!**
**LOD95%**	**9**					**15**					**15**				

Intra-run variability

**a**	-3.159				-3.144				-3.148			
**b**	37.27				35.82				36.03			
**1**	3.98	2.96	1.81	-0.11	3.76	2.82	1.59	0.24	3.73	2.79	1.53	0.15
**2**	3.92	3.09	1.92	0.69	3.74	2.99	1.76	0.72	3.74	2.97	1.71	0.78
**3**	4.04	2.95	1.73	1.00	3.86	2.82	1.63	0.85	3.87	2.77	1.62	0.79
**4**	4.03	3.06	1.80	0.14	3.84	2.91	1.61	0.13	3.81	2.93	1.64	0.25
**5**	3.98	2.98	1.72	0.81	3.75	2.87	1.54	0.69	3.75	2.89	1.50	0.68
**6**	3.96	3.16	1.74	1.14	3.76	3.03	1.58	0.97	3.79	3.04	1.54	0.97
**7**	4.00	3.04	1.84	1.00	3.79	2.91	1.71	0.82	3.82	2.90	1.75	0.86
**8**	4.04	3.14	1.89	0.91	3.83	3.03	1.65	0.78	3.85	3.06	1.64	0.77
**9**	4.00	3.05	1.79	0.84	3.83	2.90	1.60	0.79	3.89	2.85	1.59	0.74
**10**	3.98	2.97	1.87	0.96	3.79	2.87	1.71	0.84	3.81	2.85	1.72	0.90
**11**	4.00	2.96	1.97	1.10	3.78	2.86	1.76	0.95	3.76	2.83	1.72	0.92
**12**	3.95	2.92	1.87	1.07	3.78	2.76	1.75	-0.20	3.81	2.70	1.71	-0.17
**13**	3.99	3.02	1.83	0.80	3.79	2.90	1.66	0.63	3.80	2.88	1.64	0.64
**AVERAGE**	**3.99**	**3.02**	**1.83**	**0.80**	**3.79**	**2.90**	**1.66**	**0.63**	**3.80**	**2.88**	**1.64**	**0.64**
**STDEV**	**0.03**	**0.07**	**0.07**	**0.37**	**0.04**	**0.08**	**0.07**	**0.35**	**0.05**	**0.10**	**0.08**	**0.34**
**%CV**	**0.85**	**2.48**	**4.09**	**47.00**	**1.00**	**2.78**	**4.48**	**56.09**	**1.27**	**3.53**	**4.88**	**54.03**

Inter-run variability

**replicat**	**1.00E+05**	**1.00E+04**	**1.00E+03**	**1.00E+05**	**1.00E+04**	**1.00E+03**	**1.00E+05**	**1.00E+04**	**1.00E+03**
**1**	**24.76**	27.85	31.05	24.03	27.14	30.36	23.58	26.67	30.09
**2**	**24.62**	27.73	31.03	23.78	26.94	30.35	23.08	26.42	30.06
**3**	**24.6**	27.75	31.15	23.9	27.1	30.46	23.36	26.71	30.05
**1**	3.96	2.98	1.97	3.75	2.76	1.74	3.95	2.97	1.89
**2**	4.00	3.02	1.98	3.83	2.82	1.74	4.11	3.05	1.90
**3**	4.01	3.01	1.94	3.79	2.77	1.70	4.02	2.96	1.90
**AVERAGE**	3.98	2.98	1.92	3.79	2.81	1.73	3.94	3.00	1.81
**STDEV**	0.02344377	0.0373199	0.062862	0.025478	0.0577864	0.038631	0.112762	0.0626135	0.09767
**%CV**	**0.59**	**1.25**	**3.27**	**0.67**	**2.05**	**2.24**	**2.86**	**2.09**	**5.41**
